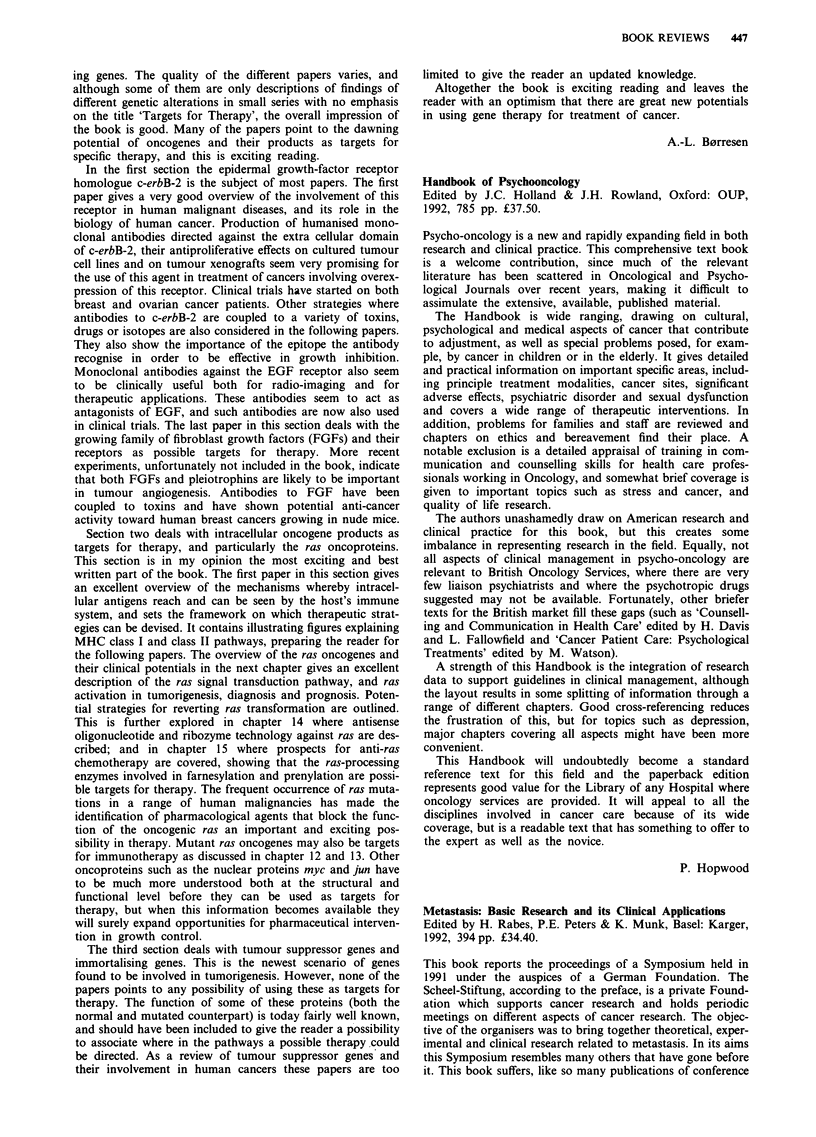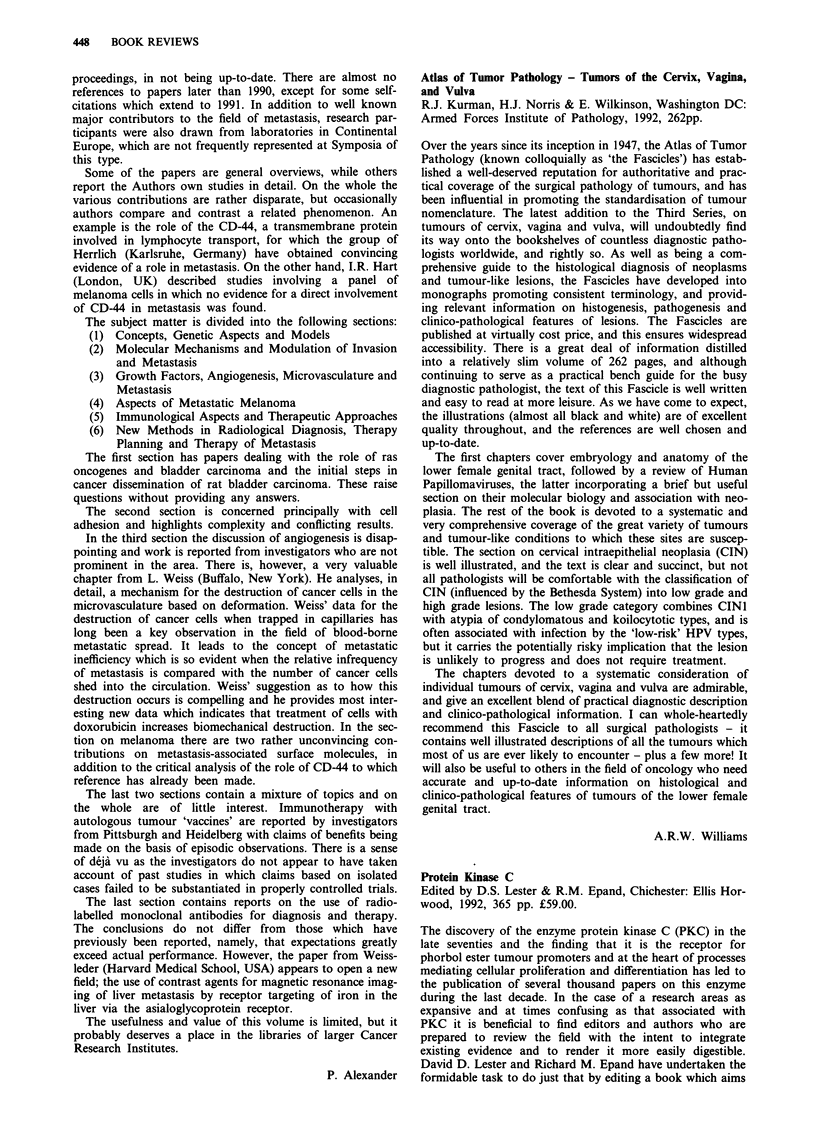# Metastasis: Basic Research and its Clinical Applications

**Published:** 1993-08

**Authors:** P. Alexander


**Metastasis: Basic Research and its Clinical Applications**
Edited by H. Rabes, P.E. Peters & K. Munk, Basel: Karger, 1992, 394 pp. £34.40.

This book reports the proceedings of a Symposium held in 1991 under the auspices of a German Foundation. The Scheel-Stiftung, according to the preface, is a private Foundation which supports cancer research and holds periodic meetings on different aspects of cancer research. The objective of the organisers was to bring together theoretical, experimental and clinical research related to metastasis. In its aims this Symposium resembles many others that have gone before it. This book suffers, like so many publications of conference proceedings, in not being up-to-date. There are almost no references to papers later than 1990, except for some self-citations which extend to 1991. In addition to well known major contributors to the field of metastasis, research participants were also drawn from laboratories in Continental Europe, which are not frequently represented at Symposia of this type.

Some of the papers are general overviews, while others report the Authors own studies in detail. On the whole the various contributions are rather disparate, but occasionally authors compare and contrast a related phenomenon. An example is the role of the CD-44, a transmembrane protein involved in lymphocyte transport, for which the group of Herrlich (Karlsruhe, Germany) have obtained convincing evidence of a role in metastasis. On the other hand, I.R. Hart (London, UK) described studies involving a panel of melanoma cells in which no evidence for a direct involvement of CD-44 in metastasis was found.

The subject matter is divided into the following sections:
(1) Concepts, Genetic Aspects and Models
(2) Molecular Mechanisms and Modulation of Invasion and Metastasis
(3) Growth Factors, Angiogenesis, Microvasculature and Metastasis
(4) Aspects of Metastatic Melanoma
(5) Immunological Aspects and Therapeutic Approaches
(6) New Methods in Radiological Diagnosis, Therapy Planning and Therapy of Metastasis

The first section has papers dealing with the role of ras oncogenes and bladder carcinoma and the initial steps in cancer dissemination of rat bladder carcinoma. These raise questions without providing any answers.

The second section is concerned principally with cell adhesion and highlights complexity and conflicting results.

In the third section the discussion of angiogenesis is disappointing and work is reported from investigators who are not prominent in the area. There is, however, a very valuable chapter from L. Weiss (Buffalo, New York). He analyses, in detail, a mechanism for the destruction of cancer cells in the microvasculature based on deformation. Weiss' data for the destruction of cancer cells when trapped in capillaries has long been a key observation in the field of blood-borne metastatic spread. It leads to the concept of metastatic inefficiency which is so evident when the relative infrequency of metastasis is compared with the number of cancer cells shed into the circulation. Weiss' suggestion as to how this destruction occurs is compelling and he provides most interesting new data which indicates that treatment of cells with doxorubicin increases biomechanical destruction. In the section on melanoma there are two rather unconvincing contributions on metastasis-associated surface molecules, in addition to the critical analysis of the role of CD-44 to which reference has already been made.

The last two sections contain a mixture of topics and on the whole are of little interest. Immunotherapy with autologous tumour 'vaccines' are reported by investigators from Pittsburgh and Heidelberg with claims of benefits being made on the basis of episodic observations. There is a sense of déjà vu as the investigators do not appear to have taken account of past studies in which claims based on isolated cases failed to be substantiated in properly controlled trials.

The last section contains reports on the use of radio-labelled monoclonal antibodies for diagnosis and therapy. The conclusions do not differ from those which have previously been reported, namely, that expectations greatly exceed actual performance. However, the paper from Weissleder (Harvard Medical School, USA) appears to open a new field; the use of contrast agents for magnetic resonance imaging of liver metastasis by receptor targeting of iron in the liver via the asialoglycoprotein receptor.

The usefulness and value of this volume is limited, but it probably deserves a place in the libraries of larger Cancer Research Institutes.

P. Alexander